# Differential pattern and prognostic significance of CD4^+^, FOXP3^+ ^and IL-17^+ ^tumor infiltrating lymphocytes in ductal and lobular breast cancers

**DOI:** 10.1186/1471-2407-12-134

**Published:** 2012-04-03

**Authors:** Raoul Droeser, Inti Zlobec, Ergin Kilic, Uwe Güth, Michael Heberer, Giulio Spagnoli, Daniel Oertli, Coya Tapia

**Affiliations:** 1 Department of Surgery, University Hospital Basel, Spitalstrasse 21, 4031 Basel, Switzerland; 2 Institute for Surgical Research and Hospital Management ICFS and Department of Biomedicine, University of Basel, Hebelstrasse 20, 4031 Basel, Switzerland; 3 Institute for Pathology, University Hospital Bern, Murtenstrasse 31, 3010 Bern, Switzerland; 4 Institute for Pathology, University Hospital Basel, Schönbeinstrasse 40, 4031 Basel, Switzerland; 5 Institute for Pathology, Charité University Hospital, Campus-Mitte, Charitéplatz 1, 10117 Berlin, Germany; 6 Department of Gynecology and Obstetrics, University Hospital Basel, Spitalstrasse 21, 4056 Basel, Switzerland

## Abstract

**Background:**

Clinical relevance of tumor infiltrating lymphocytes (TILs) in breast cancer is controversial. Here, we used a tumor microarray including a large series of ductal and lobular breast cancers with long term follow up data, to analyze clinical impact of TIL expressing specific phenotypes and distribution of TILs within different tumor compartments and in different histological subtypes.

**Methods:**

A tissue microarray (TMA) including 894 ductal and 164 lobular breast cancers was stained with antibodies recognizing CD4, FOXP3, and IL-17 by standard immunohistochemical techniques. Lymphocyte counts were correlated with clinico-pathological parameters and survival.

**Results:**

CD4^+ ^lymphocytes were more prevalent than FOXP3^+ ^TILs whereas IL-17^+ ^TILs were rare. Increased numbers of total CD4^+ ^and FOXP3^+ ^TIL were observed in ductal, as compared with lobular carcinomas. High grade (G3) and estrogen receptor (ER) negative ductal carcinomas displayed significantly (*p* < 0.001) higher CD4^+ ^and FOXP3^+ ^lymphocyte infiltration while her2/neu over-expression in ductal carcinomas was significantly (*p* < 0.001) associated with higher FOXP3^+ ^TIL counts. In contrast, lymphocyte infiltration was not linked to any clinico-pathological parameters in lobular cancers. In univariate but not in multivariate analysis CD4^+ ^infiltration was associated with significantly shorter survival in patients bearing ductal, but not lobular cancers. However, a FOXP3^+^/CD4^+ ^ratio > 1 was associated with improved overall survival even in multivariate analysis (*p *= 0.033).

**Conclusions:**

Ductal and lobular breast cancers appear to be infiltrated by different lymphocyte subpopulations. In ductal cancers increased CD4^+ ^and FOXP3^+ ^TIL numbers are associated with more aggressive tumor features. In survival analysis, absolute numbers of TILs do not represent major prognostic indicators in ductal and lobular breast cancer. Remarkably however, a ratio > 1 of total FOXP3^+^/CD4^+ ^TILs in ductal carcinoma appears to represent an independent favorable prognostic factor.

## Background

Tumor-infiltrating lymphocytes (TILs) are frequently considered to reflect host immune response against malignant tumors [1]. TILs have been shown to infiltrate a variety of tumors of diverse histological origin [2,3]. Their exquisite tumor specificity has been demonstrated in a number of cases and it has led to the characterization of tumor associated antigens. Although resident TILs have frequently been reported to be in a functionally "anergic" state [4,5], importantly, following "*ex vivo*" culture, TILs have been used to treat different types of cancers [6]. In line with these data, tumor infiltration by T lymphocytes has been shown to be associated with favorable prognosis, particularly in melanoma and colorectal cancers [2,7]. On the other hand, tumor infiltration by T-lymphocytes subsets endowed with immuno-regulatory or suppressive potential, e.g. CD4^+ ^T-cells expressing FOXP3 transcription factor, has been suggested to be associated with tumor progression and unfavorable prognosis [8]. More recently, a CD4^+ ^T-cell subset producing IL-17 has been implicated in the pathogenesis of several autoimmune diseases [9]. However, the role of the so-called Th17 in antitumor immunity is still debated [10-13]

In normal breast tissue small numbers of lymphocytes representing the mucosa-associated lymphoid tissue can be detected [14]. In contrast, increased numbers of lymphocytes are frequently detectable around and within breast cancers [15-18]. The clinical significance of TILs in breast cancer is still controversial. In some studies, TILs were associated with unfavorable characteristics such as high grade tumors, estrogen receptor negativity, basal-like molecular subtype as well as her2/neu positive tumors [19,20]. High CD4^+ ^and CD8^+ ^lymphocytic infiltration has been associated with positive lymph node status as well as worse overall survival [21]. Furthermore, in early stage breast cancer, CD8^+ ^lymphocytic infiltration has been suggested to correlate with lymph node involvement [22]. Other groups, however, have shown that breast cancers with increased TIL number display a better prognosis in comparison with breast cancers with lesser lymphocyte infiltration [23], as also confirmed by data from our institution for CD8^+ ^TILs in the ER negative subgroup [24]. Additionally, high TIL counts may represent an independent predictor of response to neo-adjuvant chemotherapy [25]. Notably, infiltration by FOXP3^+ ^lymphocytes in breast cancer has been proposed to represent an independent unfavorable prognostic factor, especially in the nodal positive subgroup [26] and to correlate with tumor invasiveness [27]. In contrast, a complete clinical response has been suggested to be associated with disappearance of tumor infiltrating FOXP3^+ ^T-cells during treatment [28].

While the clinical significance of TILs is controversial, their distribution within stromal and intratumoral compartments in breast cancers is largely unknown. Furthermore, T-cell infiltration in different histological subtypes as well as the occurrence of IL-17^+ ^lymphocytes in breast cancer tissue has not been reported to date. Here, we addressed these issues by using a tissue microarray (TMA) including a large number (> 1000) of breast cancers stratified according to ductal and lobular histological subtypes. By taking advantage of a comprehensive clinical follow-up database, numbers of CD4^+^, FOXP3^+ ^and IL-17^+ ^TILs and their occurrence in different tumor compartments was correlated with clinico-pathological features and survival data.

## Methods

### Breast cancer tissue microarray

The TMA utilized in this study includes 894 (84.5%) invasive ductal and 164 (15.5%) lobular paraffin-embedded breast cancers diagnosed at the Institute for Pathology, University Hospital of Basel and at the Viollier Institute in Basel between 1985 and 2007. For 386 (36%) tumors, punches from center and periphery of cancerous tissues were available. The median age of patients was 63 years (range 28-94). The mean follow-up time was 84.5 months (median: 75 months; range 1-263 months). Raw patient survival data were obtained from the Cancer Registry of Basel or from the patients' attending physicians. TNM classification and tumor diameter were obtained from pathology reports. Hormone receptor and her2/neu status data were available from a previous study from our group [29]. TMA were dressed with materials from the Tissue Biobank of the Institute of Pathology, University Hospital Basel. This institution performs translational research with approval of the EKBB (ethical committee Beider Basel) in compliance with and specific regard to ethical standards and patient confidentiality. The clinico-pathological characteristics of the patients are listed in Table 1. Micro-invasive carcinomas and carcinomas of T4 stage were excluded from this study since microinvasion can hardly be punched on a TMA and T4 breast cancers represent a heterogeneous subgroup with ambiguous classification criteria and different prognosis [30].

**Table 1:** Clinico-pathological characteristics of patient cohort

Feature		Frequency n (%)	
		**Ductal carcinoma (n = 894)**	**Lobular carcinoma (n = 164)**

**pT stage**	pT1	369 (41.3)	52 (31.7)
	
	pT2	475 (53.1)	90 (54.9)
	
	pT3	50 (5.6)	22 (13.4)

**pN stage**	pN0	485 (54.3)	90 (54.9)
	
	pN1	340 (38.0)	67 (40.8)
	
	pN2	69 (7.7)	7 (4.3)

**Clinical stage**	IA	254 (28.4)	39 (23.8)
	
	IIA	325 (36.4)	54 (32.9)
	
	IIB	211 (23.6)	42 (25.6)
	
	IIIA	104 (11.6)	29 (17.7)

**BRE grade**	G1	181 (20.4)	24 (14.7)
	
	G2	372 (41.9)	118 (72.4)
	
	G3	334 (37.7)	21 (12.9)

**ER status**	Negative	200 (23.7)	22 (14.4)
	
	Positive	645 (76.3)	131 (85.6)

**PR status**	Negative	346 (52.6)	65 (54.6)
	
	Positive	312 (47.4)	54 (45.4)

**Her-2/neu**	0 and 1+	680 (83.1)	130 (94.2)
	
	2+ and 3+	138 (16.9)	8 (5.8)

**Molecular Subtypes**	Luminal A	160 (40.1)	30 (48.4)
	
	Luminal B	7 (1.8)	0 (0.0)
	
	Her2/neu	64 (16.0)	3 (4.8)
	
	Triple Negative	168 (42.1)	29 (46.8)

**Disease-specific survival**	death from breast cancer	72 (8.7)	9 (5.8)
	
	Censored	760 (91.3)	145 (94.2)
	
	5-year (95%CI)	94.0 (92-96)	97.0 (92-99)

**Overall survival**	Death	172 (19.4)	34 (21.3)
	
	Censored	714 (80.6)	126 (78.7)
	
	5-year (95%CI)	85.6 (83-88)	87.8 (81-92)

**Age at diagnosis (y)**	median	62	64
	
	Minimum-maximum	28-94	38-90

The TMA was constructed as previously described [31]. Briefly, formalin-fixed, paraffin-embedded tissue blocks of breast cancer samples were obtained. Tissue cylinders with a diameter of 0.6 mm were punched from morphologically representative cancer areas of a donor tissue block and brought into a recipient paraffin block (30 × 25 mm), using a semiautomated tissue arrayer.

### Immunohistochemistry (IHC) and evaluation

Four-micron sections of the TMA blocks were incubated with the following primary antibodies at the indicated dilutions: mouse anti-human CD4 antibody (clone 1F6, Novocastra Labs, Newcastle. UK), undiluted (RU). Monoclonal mouse anti-human FOXP3 (clone 236A/E7, Abcam, Cambridge, UK), dilution: 1:20; purified polyclonal goat anti-human IL-17 antibody (R&D Systems, Minneapolis, Minnesota, USA), dilution 1:50; purified polyclonal anti-human CD3 (Dako, Glostrup, Denmark), dilution 1:600; monoclonal anti-human CD163; (clone 10D6, NeoMarkers, Fremont, CA), dilution 1:40.

**Figure 1:**
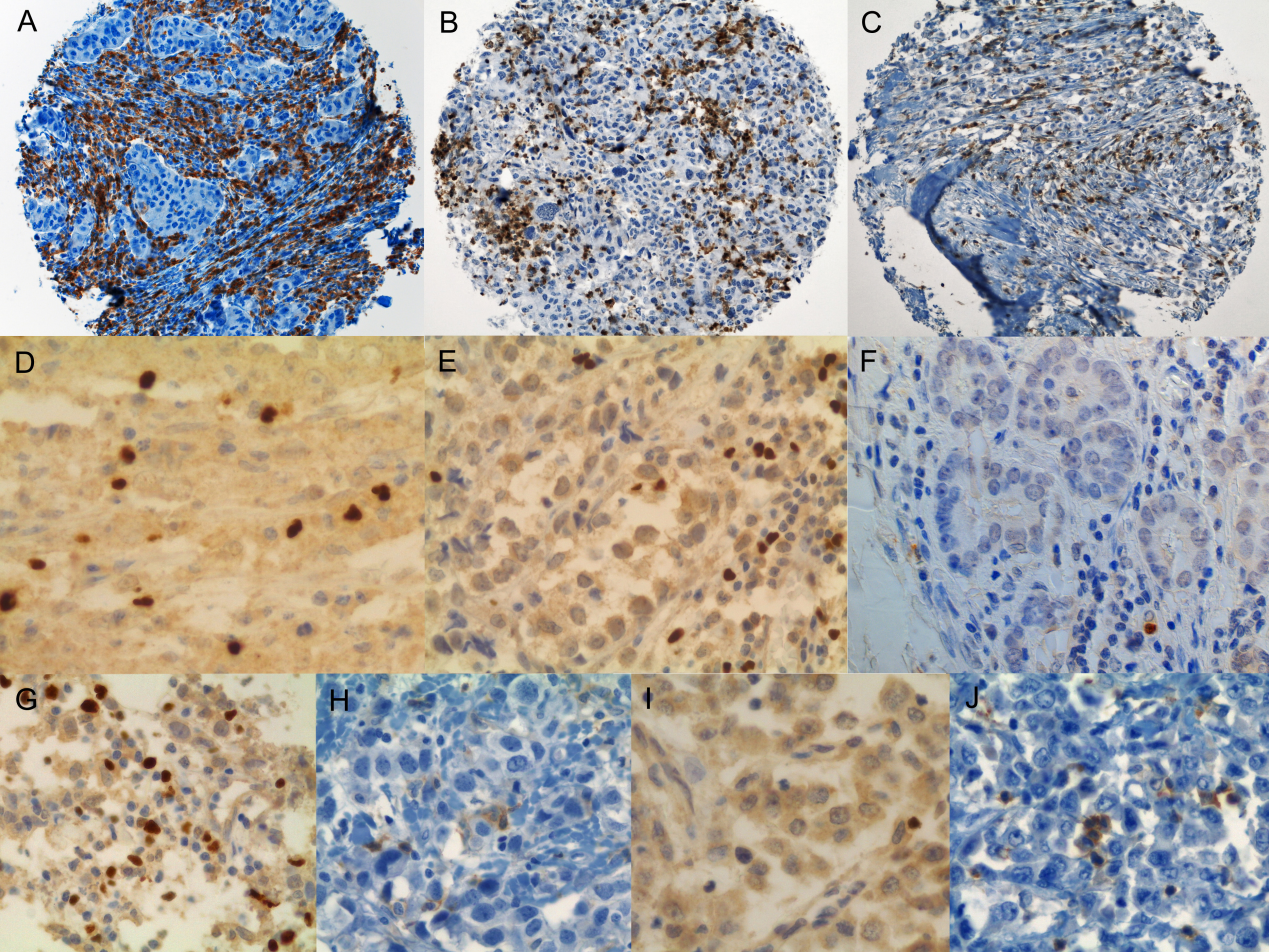
**Examples of CD4^+^, FOXP3^+ ^and IL-17^+ ^infiltrating lymphocytes in ductal and lobular breast cancer**. A, B, C: Immunohistochemical staining of breast cancer tissue punches incubated with anti-CD4 antibody (brown staining): A: Ductal breast cancer with predominantly stromal and intratumoral CD4^+ ^cells. B: Ductal breast cancer with prominent cell pleomorphism and easily detectable intratumoral CD4^+ ^cells, within the tumor and surrounding it. C: Example of an invasive lobular breast cancer with CD4^+ ^cells. D; E: Zoom of breast cancers incubated with anti-FOXP3 antibody (brown staining): D: Invasive ductal breast cancer with clear intratumoral infiltration by FOXP3^+ ^cells. E: Invasive lobular breast cancer with stromal and intratumoral FOXP3^+ ^cells. F: Ductal breast cancer showing a single infiltrating IL-17^+ ^cell (brown staining). G/H: Ductal carcinoma with a FOXP3/CD4 ratio > 1. G shows the FOXP3 staining and H the CD4 staining. I/J: Lobular cancer with a FOXP3/CD4 ratio < 1. I shows the FOXP3 staining and J the CD4 staining.

**Figure 2:**
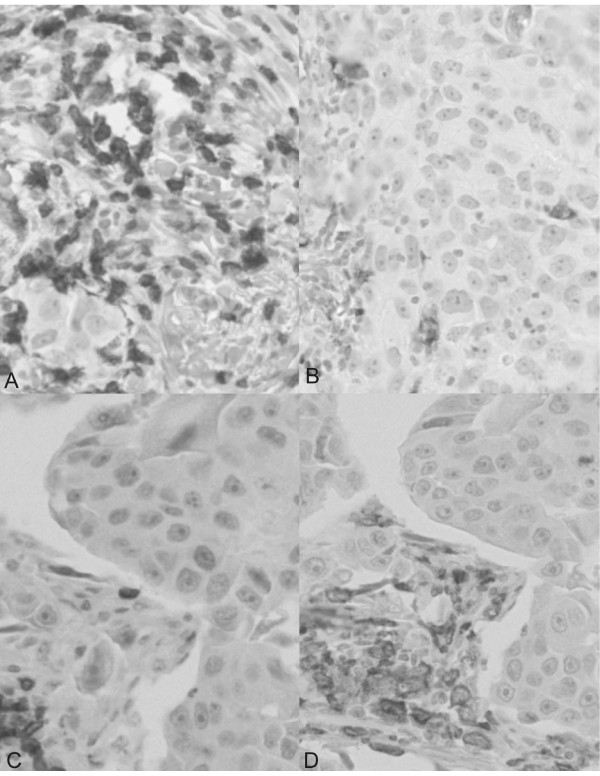
**Examples of CD3 and CD163 specific staining**. Immunohistochemistry of invasive ductal breast cancer infiltrated by CD3^+ ^(A, C) and CD163^+ ^(B, D) cells (brown staining) in tumor samples with FOXP3/CD4 ratios > 1 (A, B) and < 1 (C, D).

Numbers of stained TILs were counted in each tissue spot, representing approximately one 40x high-power-field. Lymphocytes in contact or within the tumor epithelium were scored as "intratumoral" whereas lymphocytes in the interstitial space were defined as "stromal" (Figures 1 and 2).

### Statistical analysis section

Differences in histo-pathological features between ductal and lobular breast cancers were analyzed using Chi-Square or Fisher's Exact tests, while differences in the number of TILs were investigated by using the non-parametric Wilcoxon Rank Sum test. Univariate survival analysis was carried out with the log-rank test and prognostic differences were illustrated using Kaplan-Meier curves. Cut-off scores for high and low TILs were determined using receiver operating characteristic (ROC) curve analysis. Additionally, multiple Cox regression analysis was performed to test the independent prognostic effect of TILs on outcome after adjusting for well-established prognostic factors. The assumption of proportional hazards was tested and validated by evaluating the log(-log(survival)) versus log of survival time graphs. Finally, correlations between different cell populations were evaluated using Spearman's rank correlation coefficient. In order to resolve the issue of multiple hypothesis testing, a Bonferroni correction for multiple comparisons was undertaken; hence p-values < 0.001 were considered statistically significant while values < 0.05 were identified as trends. Although the Bonferroni correction is considered highly conservative, it was used here to ensure a high level of confidence with regard to the rejection of the null hypothesis. Analyses were performed using SAS (V9.1, the SAS Institute, Cary, NC).

## Results

### Lymphocyte infiltration in breast cancer subtypes

**Figure 3:**
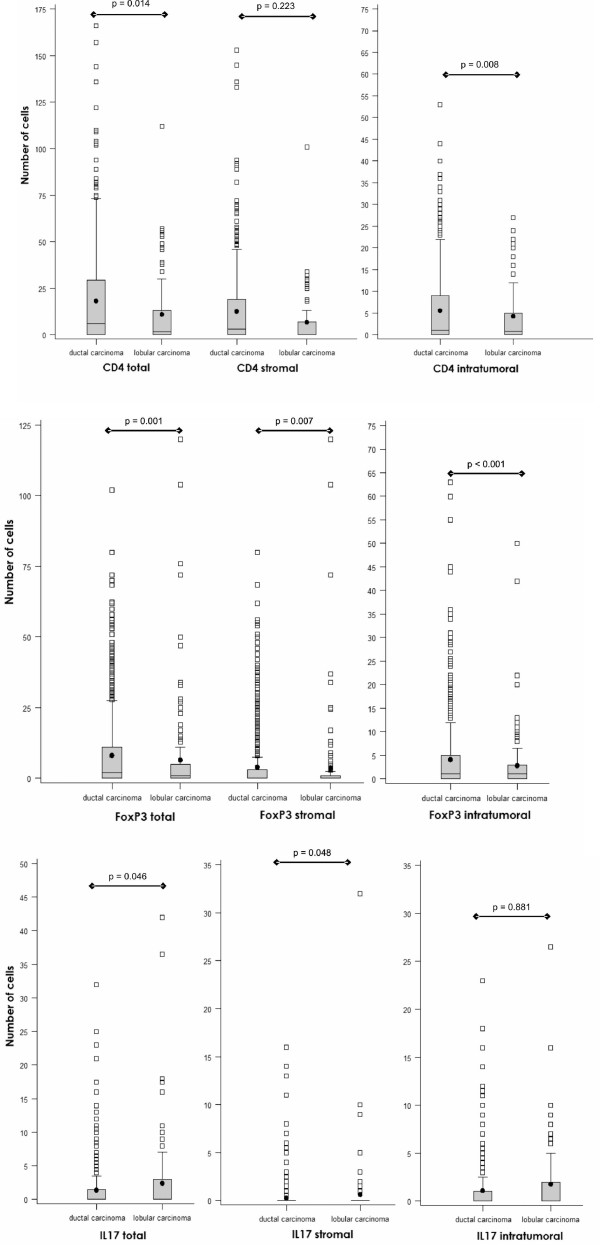
**Box plot analysis of CD4^+^, FOXP3^+ ^and IL-17^+ ^cell distribution in ductal versus lobular carcinoma**. Box plot analysis of lymphocyte infiltration in ductal versus lobular carcinoma for CD4^+^, FOXP3^+ ^and IL-17^+ ^TILs. After adjustment for multiple comparisons, only *p *< 0.001 was considered statistically significant.

Initially, we addressed overall lymphocyte infiltration in breast cancer subtypes. Ductal breast cancers displayed a higher CD4^+ ^lymphocyte total (18.2 ± 25.3 vs. 11.1 ± 19.4; *p *= 0.014) and intratumoral infiltration as compared to lobular tumors (12.6 ± 20.3 vs. 6.8 ± 14.1, *p *= 0.008). Infiltrating FOXP3^+ ^cells were detectable to a significantly higher extent within the tumor (4.0 ± 9.1 vs. 3.7 ± 14.4, *p *= 0.001) and in the stroma (4.2 ± 1.4 vs. 2.8 ± 6.1, *p *= 0.007) of ductal cancers as compared to lobular breast cancers (Figure 3).

Remarkably, there was a significant correlation between total CD4^+ ^and FOXP3^+ ^cells (*p* < 0.001; r = 0.47). This correlation was maintained in both ductal and lobular carcinomas.

Only very few IL-17^+ ^lymphocytes were detected in either cancer subtype (ductal: 1.4 ± 3.0; lobular: 2.4 ± 5.4, *p *= 0.046), with a slight trend towards relatively higher counts in the stromal compartment of lobular as compared to ductal carcinomas (1.1 ± 2.3 vs. 1.8 ± 3.4; *p *= 0.048).

Analysis of specimens (n = 386) for which two punches from tumor center and periphery, respectively, were available, did not show any significant difference in infiltrate numbers for none of the markers under investigation: CD4 total (*p *= 0.951), CD4 intratumoral (*p *= 0.845), CD4 stromal (*p *= 0.297), FOXP3 total (*p *= 0.175), FOXP3 intratumoral (*p *= 0.11), FOXP3 stromal (*p *= 0.916), IL17 total (*p *= 0.292), IL-17 intratumoral and (*p *= 0.228) and IL17 stromal (*p *= 0.492).

### High grade ductal carcinomas are associated with increasing numbers of CD4^+ ^and FOXP3^+ ^lymphocytes

Next, the correlation between histological grading and tumor infiltration was evaluated. In ductal carcinomas, a significant (*p* < 0.001) association between higher total and intratumoral numbers of CD4^+ ^and FOXP3^+ ^lymphocytes and histological grade [32] could be observed (Table 2). In contrast, regarding lobular breast cancer no association between lymphocyte infiltration and tumor grade was detectable.

**Table 2:** Correlation of CD4^+ ^and FOXP3^+ ^TILs and B.R.E. grade of ductal carcinomas

	Ductal carcinoma			
**Compartment/TIL subtype**	**B.R.E. grade**			

	**1**	**2**	**3**	p-value*

**Total**				

**CD4^+^**	10.9 ± 21.2	18.0 ± 24.3	21.2 ± 26.2	** < 0.001**

**FOXP3^+^**	4.8 ± 10.7	7.0 ± 11.4	11.2 ± 15.6	** < 0.001**

**Intratumoral**				

**CD4^+^**	7.5 ± 17.2	12.3 ± 20.1	14.8 ± 20.2	** < 0.001**

**FOXP3^+^**	1.8 ± 5.8	3.1 ± 7.2	6.1 ± 11.8	** < 0.001**

Mean ± SD, counts of immunoreactive lymphocytes CD4^+ ^and FOXP3^+ ^Wilcoxon Rank Sum Test; *after adjustment for multiple comparisons, only *p* < 0.001 was considered statistically significant

### Lymphocyte infiltration and expression of estrogen and progesterone receptors and her2/neu

The association of the prognostic and predictive markers estrogen and progesterone receptors (ER and PR), and her2/neu expression with infiltration by different T-cell subsets was then explored.

Significantly (*p* < 0.001) higher numbers of total and intratumoral CD4^+ ^and FOXP3^+ ^TILs were detected in ER negative as compared to positive ductal carcinomas.

Loss of PR expression was associated with decreased numbers of CD4^+ ^T cells in ductal carcinomas (*p *= 0.008). However, no differences regarding FOXP3^+ ^TILs according to PR expression was seen. Her2/neu over-expression was significantly (*p* < 0.001) associated with increased numbers of tumor infiltrating FOXP3^+ ^T cells and by a similar trend for CD4^+ ^lymphocytes (*p *= 0.023). These results are summarized in Table 3.

**Table 3:** Correlation of CD4^+ ^and FOXP3^+ ^TILs with hormone receptor and her2/neu status in ductal carcinomas

Compartment/TIL subtype	ER status			PR status			Her2/neu		
	**-**	**+**	**p-value**	**-**	**+**	**p-value**	**-**	**+**	**p-value**

**Total**									

**CD4^+^**	25.1 ± 29.7	15.6 ± 22.9	** < 0.001**	12.4 ± 19.3	17.9 ± 25.2	**0.008**	17.0 ± 24.5	22.4 ± 27.4	**0.023**

**FOXP3^+^**	11.9 ± 16.1	6.9 ± 11.3	** < 0.001**	8.6 ± 13.4	6.7 ± 10.9	**0.203**	7.6 ± 12.6	10.6 ± 13.0	** < 0.001**

**Intratumoral**									

**CD4^+^**	17.5 ± 23.4	10.7 ± 18.5	** < 0.001**	8.2 ± 13.6	12.7 ± 21.6	**0.032**	11.7 ± 19.7	15.6 ± 21.8	**0.036**

**FOXP3^+^**	7.0 ± 12.8	3.0 ± 7.0	** < 0.001**	4.0 ± 9.2	2.7 ± 6.1	**0.12**	3.7 ± 8.7	4.7 ± 8.1	**0.009**

Mean ± SD, counts of immunoreactive lymphocytes CD4^+ ^and FOXP3^+ ^Wilcoxon Rank Sum Test; *after adjustment for multiple comparisons, only *p* < 0.001 was considered statistically significant

On the other hand, in lobular type of breast cancer no association between hormone receptor status and distribution of TIL was observed. All her2/neu over-expressing lobular carcinomas regardless of their hormone receptor status showed an increased infiltration by intratumoral FOXP3^+ ^cells (*p *= 0.002).

Notably, triple negative ductal carcinomas showed significantly higher numbers of intratumoral (*p* < 0.001) FOXP3^+ ^TILs. The molecular subtype of her2/neu over-expressing ductal breast cancers (ER-, PR-, her2/neu+) showed an increased stromal (*p *= 0.003) and total (*p *= 0.002) number of FOXP3^+ ^lymphocytes as compared to the other molecular subtypes such as luminal A (ER+, PR+, her2/neu-) or luminal B (ER+, PR+, her/neu2+). In contrast, CD4^+ ^T-cells infiltration did not appear to be significantly associated with these molecular subgroups.

Once more, no significant association was detectable in tumors of the lobular subtype. However, an association of increased numbers of FOXP3^+ ^intratumoral cells could be found in the her2/neu over-expressing molecular subtype (ER-, PR-, her2/neu+) of lobular breast cancers (*p *= 0.006).

### Lymphocyte infiltration and breast cancer staging

Higher total numbers of FOXP3^+ ^(*p *= 0.026) lymphocytes were observed in small breast cancers (tumor size ≤ 2 cm; T1) with positive lymph nodes (N+), as compared with tumors of the same T1 stage without lymph node metastases (N0). However, in multivariate analysis, infiltration by FOXP3^+ ^cells did not represent an independent factor when adjusted for stage. Notably, an overall trend (*p *= 0.02) suggesting an association between total number of FOXP3^+ ^TIL and higher tumor stage could be observed (Stage IA: 7.1 ± 13.6, Stage IIA: 7.9 ± 13.3, Stage IIB: 7.9 ± 13.7 and Stage: IIIA: 9.5 ± 15.8).

### Lymphocyte infiltration and overall survival in patients with breast cancer

**Figure 4:**
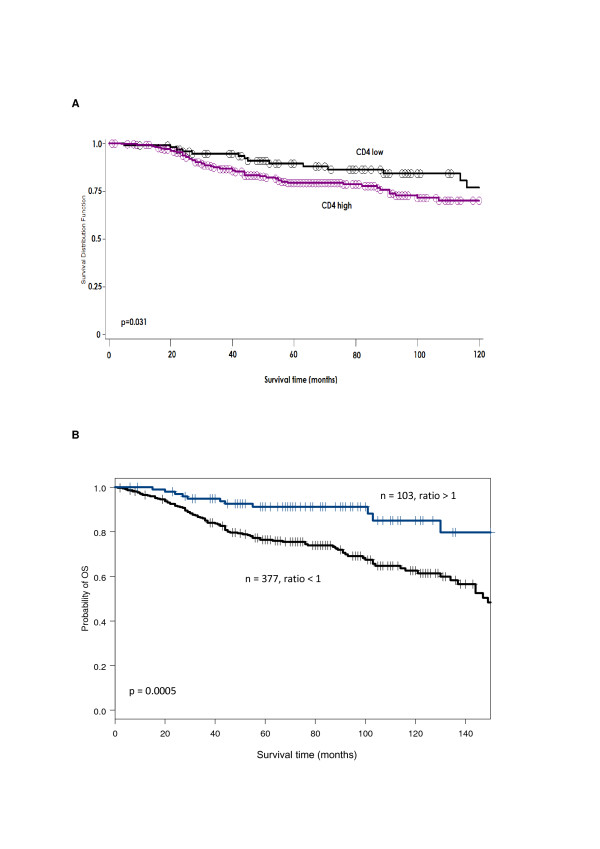
**Overall survival analysis in patients with ductal breast cancer**. A: Overall survival in patients with ductal carcinomas and high and low level of CD4^+ ^TILs. Kaplan-Meier survival curves (univariate-analysis) of ductal breast cancers and overall survival in patients with high level of CD4^+ ^versus low level of CD4^+ ^cells infiltrating the tumor. CD4^+ ^infiltrating cells: high level of CD4^+ ^violet curve; low level of CD4^+ ^black curve. Patients with high level of CD4^+ ^cell infiltration show a significant (*p *= 0.031) worse survival (cut-off established by ROC curves: > 6 CD4^+ ^TILs). B: Overall survival in patients with ductal carcinomas according to total FOXP3/CD4 ratio > or < 1. Kaplan-Meier survival curves (univariate-analysis) of ductal breast cancers and overall survival in patients with ratio of total FOXP3^+^/CD4^+ ^cells > 1 (blue curve) or < 1 (black curve). Patients with a > 1 ratio had a significantly better survival (*p *= 0.0005).

Patients with ductal breast cancers infiltrated by high total numbers of CD4^+ ^lymphocytes showed a significantly (*p *= 0.031) worse overall survival, as compared to patients with lower CD4^+ ^infiltration (Figure 4A).

Multivariate analysis, however, indicated that infiltration by CD4^+ ^did not represent an independent prognostic marker. In patients bearing lobular breast cancers survival was not significantly associated with infiltration by any lymphocyte subtype.

For 480 ductal and for 72 lobular carcinoma samples FOXP3^+^/CD4^+ ^cell ratios and overall survival data were available. In ductal carcinoma, a FOXP3^+^/CD4^+ ^> 1 was significantly associated with better survival (*p *= 0.0005, Figure 4B). This beneficial effect was maintained in a multivariate analysis (n = 352) including pT, pN, BRE grade, ER status and PR status (*p *= 0.033). In contrast, in lobular carcinoma FOXP3^+^/CD4^+ ^ratio was not significantly associated with survival.

In order to obtain insights into the nature of FOXP3^+ ^cells associated with favorable prognosis in ductal carcinoma, TMA slides were stained with anti CD3 and anti CD163 Abs (Figure 2). We observed a higher tumor infiltration by CD3 positive cells (average: 37.9 per punch) than by CD163 positive cells (average: 21.6 per punch). Most importantly, total FOXP3^+ ^cell counts were highly correlated with total numbers of CD3^+ ^cells (r = 0.503), and, in particular, with total CD8^+ ^cell numbers (r = 0.507), but much less with total numbers of CD163^+ ^cells (r = 0.321).

## Discussion

The adaptive immune system is known to play a major role in the control of tumor progression in different types of cancer. Indeed, tumor infiltration by CD8^+ ^T-cells has been shown to represent an important prognostic factor in melanoma [7], and, more recently, in colorectal cancers [2,33].

Early studies have suggested a favorable prognostic effect of lymphocyte infiltration in breast cancers [34]. More recently, infiltration by CD3^+ ^T-cells has been suggested to predict responsiveness to neoadjuvant treatment in these tumors [25]. Furthermore, a predictive effect of breast cancer infiltration by FOXP3^+ ^cells has also been reported [35]. Breast cancers, however, comprise histologically different tumor entities characterized by molecular specificities and differential prognosis [36,37]. The expression of hormone receptors and her2/neu also represent important factors in the biology of breast cancers and in its prognosis [36]. Most importantly, phenotypes and location of tumor infiltrating lymphocytes are emerging as important issues in cancer immunobiology [7]. Therefore, a thorough assessment of the immunobiological relevance of lymphocyte infiltration in breast cancer needs to accurately take into account these parameters.

Our data, deriving from the study of a large cohort of cases, including > 1000 specimens, are consistent with a significantly different pattern of lymphocyte infiltration in ductal and lobular breast cancers. In the lobular histological type, there is a lower lymphocytic infiltration than in ductal cancers, particularly regarding CD4^+ ^and FOXP3^+ ^cells. Furthermore, in lobular breast cancers, lymphocyte infiltration is not correlated with tumor grade and expression of hormonal receptors and it has no prognostic relevance.

In contrast, in ductal cancers, increased infiltration by CD4^+ ^or FOXP3^+ ^lymphocytes correlates significantly with histological grade, and ER loss. Her2/neu over-expression in ductal cancers is also significantly associated with increased numbers of FOXP3^+ ^infiltration. Puzzlingly, loss of PR expression appears to be associated with a decrease of CD4^+ ^infiltrate. In agreement with previous reports [27,38], we also found that FOXP3^+ ^infiltration is significantly increased in triple negative ductal, but not in lobular breast cancers.

Whereas the molecular background underlying these effects is unclear, they do impact the clinical course of the disease, since in ductal cancers, high infiltration by CD4^+ ^T-cells is associated with a significantly more severe prognosis, albeit only in univariate analysis.

On the other hand, in both ductal and lobular cancers only a modest infiltration by IL-17 producing cells was detectable.

Ductal breast cancers are more compactly growing tumors, sometimes characterized by a broad and pushing border, whereas lobular carcinomas mainly show indian file pattern, smoothly infiltrating the surrounding tissue. Interestingly, the detection of increasing numbers of TILs in high grade tumors with pushing borders was described earlier in medullary breast cancers [23]. In our study, higher counts of TILs are significantly (*p* < 0.001) associated with more aggressive tumor features such as loss of estrogen receptor, higher tumor grade (G3), or her2/neu over-expression in ductal breast cancers. More aggressive tumors are growing faster and may therefore present more necrotic areas while producing stroma damage possibly related to local hypoxia [39-42]. Indeed, increased numbers of CD4^+ ^and FOXP3^+ ^cells under hypoxic condition could be shown in several studies [43]. Therefore, the growth dynamics of the tumor could play a role in inducing lymphocyte infiltration.

FOXP3 represents a typical, although not exclusive, marker of regulatory CD4^+ ^T-cells. Tumor infiltration by T-cells expressing this marker has been associated to severe prognosis in different tumors, including ovarian and lung cancers [8]. However, in colorectal cancers FOXP3^+ ^T-cell infiltration has been found to correlate with significantly improved prognosis by us and others [44,45]. Our data clearly document that, although numbers of FOXP3^+ ^lymphocyte infiltration in ductal breast cancer are significantly associated with unfavourable clinico-pathological features, this marker alone does not appear to represent a prognostic marker. However, a high ratio (> 1) of total FOXP3^+^/CD4^+ ^TILs was independently associated with a better overall survival, thereby suggesting that FOXP3^+ ^cells other than CD4^+ ^T lymphocytes could be involved in the elicitation of the favorable prognostic effects. It is of note, that although FOXP3 still represents one of the most reliable Treg markers, it is known to be expressed by activated T-cells as well [8]. Indeed, FOXP3 has been shown to be expressed, albeit transiently, in activated CD8^+ ^T cells [46], in tonsillar suppressive CD8^+ ^T cells [47], and even in tumor cells [48].

Contrasting data have been reported regarding FOXP3 expression in tumor cells. In a mouse model FOXP3 could be identified as a X-linked tumor suppressor gene in breast cancer [49], but others have failed to detect expression of this gene in non hematopoietic tissues [50]. FOXP3 expression has also been reported in human breast cancer cells [26,51]. Other groups however, did not confirm these findings and only detected FOXP3 expression in breast cancer infiltrating lymphocytes [35,52]. Furthermore evidence has been reported that localization and activation status of FOXP3 positive cells might play a prognostic role in breast cancer [53,54]. Still unclear is the background underlying these discrepancies, possibly related to the use of different reagents and staining protocols. In our studies however, in keeping with previous results [55] expression of FOXP3 in breast cancer cells was found to be negligible while it was usually detectable as nuclear staining of TIL.

Accordingly, CD3 and CD163 specific staining data indicate that tumor infiltration by FOXP3^+ ^cells is highly correlated with infiltration by CD3^+ ^cells. Further studies are warranted to clarify nature, origin and functions of FOXP3^+ ^cells associated with improved survival in ductal breast cancer. Nevertheless, our data suggest that FOXP3 expression might reflect an activated state of specific T cell subsets.

Our study indicates that ductal and lobular breast cancers are characterized by a significantly different pattern of lymphocyte infiltration. Notably, in ductal cancers, total and, in particular, intratumoral lymphocyte infiltration is significantly associated with higher histological grade and severe prognosis, although not independently from known prognostic factors.

Further research is warranted to clarify whether these features are related to differential growth patterns of these tumor types, or to a differential immunogenicity of these tumors. Alternatively, variable tumor microenvironments might differentially favour lymphocyte chemoattraction and expansion.

## Conclusions

Our data, deriving from a large database, document that breast cancers may be differentially infiltrated by lymphocytes depending on their histological subtype. Furthermore, they indicate that tumor infiltration by CD4^+ ^or FOXP3^+ ^cells is devoid of prognostic relevance. Intriguingly however, they also suggest that breast cancer infiltration by CD4^-^/FOXP3^+ ^lymphocytes might represent an independent favorable prognostic factor.

## Abbreviations

ER: Estrogen receptor; FOXP3: Forkhead box P3; her2/neu: Human epidermal growth factor receptor 2; PR: Progesterone receptor; ROC: Receiver operating characteristic; TILs: Tumor-infiltrating lymphocytes; TMA: Tissue microarray; Treg: Regulatory T cell; IL-17: Interleukin-17.

## Competing interests

The authors declare that they have no competing interests.

## Authors' contributions

RD contributed to the study design, IHC evaluation and drafted the manuscript. IZ performed statistical analyses and was involved in revising the manuscript. UG collected data and contributed to the manuscript content. EK contributed to the manuscript and supervised the experiments. OD, HM, SG, contributed intellectually to the manuscript content. CT conceived the study, supervised the experiments, revised the manuscript for important intellectual content and gave final approval of the version to be published. All authors read and approved the manuscript.

## Pre-publication history

The pre-publication history for this paper can be accessed here:

http://www.biomedcentral.com/1471-2407/12/134/prepub
